# Case Report: Re-Sensitization to Gefitinib in Lung Adenocarcinoma Harboring EGFR Mutation and High PD-L1 Expression After Immunotherapy Resistance, Which Finally Transform Into Small Cell Carcinoma

**DOI:** 10.3389/fonc.2021.661034

**Published:** 2021-06-24

**Authors:** Xiaoqian Zhai, Jiewei Liu, Zuoyu Liang, Zhixi Li, Yanyang Liu, Lin Huang, Weiya Wang, Feng Luo

**Affiliations:** ^1^ Lung Cancer Center, West China Hospital, Sichuan University, Chengdu, China; ^2^ Pathology Department, West China Hospital, Sichuan University, Chengdu, China

**Keywords:** immunotherapy resistance, targeted therapy resistance, epidermal growth factor receptor mutation, high PD-L1 expression, tyrosine kinase inhibitors, lung adenocarcinoma, small cell cancer transformation

## Abstract

The treatment sequence of immunotherapy (IO) and epidermal growth factor receptor (EGFR) tyrosine kinase inhibitors (TKIs) is of great importance for the survival of non-small cell lung cancer (NSCLC) patients with EGFR sensitive mutation. Here, we reported an advanced lung adenocarcinoma case concurrent with EGFR sensitive mutation and high PD-L1 expression (>50%) that was administrated with gefitinib firstly, and then became resistant to EGFR-TKI. He received the strategy of immunity-combined chemo-radiotherapy and responded significantly. However, the disease re-progressed after 10 months. Surprisingly, the tumor re-sensitized to gefitinib for 13 months. At final, following the treatment pressure of TKI-IO combination therapy-TKI strategy, tumor clone eventually transformed into small cell lung carcinoma (SCLC). For one thing, our study provided novel approach and extended the treatment spectra of overcoming immunotherapy resistance after EGFR resistance in driver oncogene-mutated NSCLC. For another thing, our case is the first time to report that SCLC transformation can be achieved after gefitinib–pembrolizumab–gefitinib resistance in EGFR sensitive mutation NSCLC, providing a new condition for SCLC transformation.

## Background

Immune checkpoint inhibitors (ICIs) and targeted therapy have revolutionized the therapy landscapes of non-small cell lung cancer (NSCLC) which is the leading cause of cancer death worldwide. The treatment sequence of immunotherapy (IO) and epidermal growth factor receptor (EGFR) tyrosine kinase inhibitors (TKIs) is of great importance for survival of NSCLC patients with EGFR sensitive mutation. There are a few therapies having been approved in driver oncogene-mutated NSCLC after kinase inhibitor resistance. One of the study, IMpower 150, suggested the addition of atezolizumab to bevacizumab and chemotherapy (ABCP) increased PFS benefit for EGFR-TKI-resistant patients when compared with chemotherapy alone (1). However, a significant proportion of patients eventually failed to respond to ICI therapy due to the evolution of secondary resistance (2). As such, the potential treatment strategies when IO combination therapy resistance occurred sequentially after TIK resistance are still lacking. Here, we reported a lung adenocarcinoma case concurrent with EGFR sensitive mutation and high PD-L1 expression (>50%) that progressed with gefitinib, re-progressed after immune-combined chemoradiotherapy, then re-sensitized to gefitinib. Finally, following the treatment pressure of TKI-IO combination therapy-TKI strategy, tumor clone eventually transformed into small cell lung carcinoma (SCLC). For one thing, our study provided a novel approach and extended the treatment spectra of overcoming immunotherapy resistance after EGFR resistance in driver oncogene-mutated NSCLC. For another thing, our case is the first time to report that SCLC transformation can be achieved after gefitinib–embrolizumab–gefitinib resistance in EGFR sensitive mutation NSCLC, providing a new condition for SCLC transformation.

## Case Presentation

**Figure 1:**
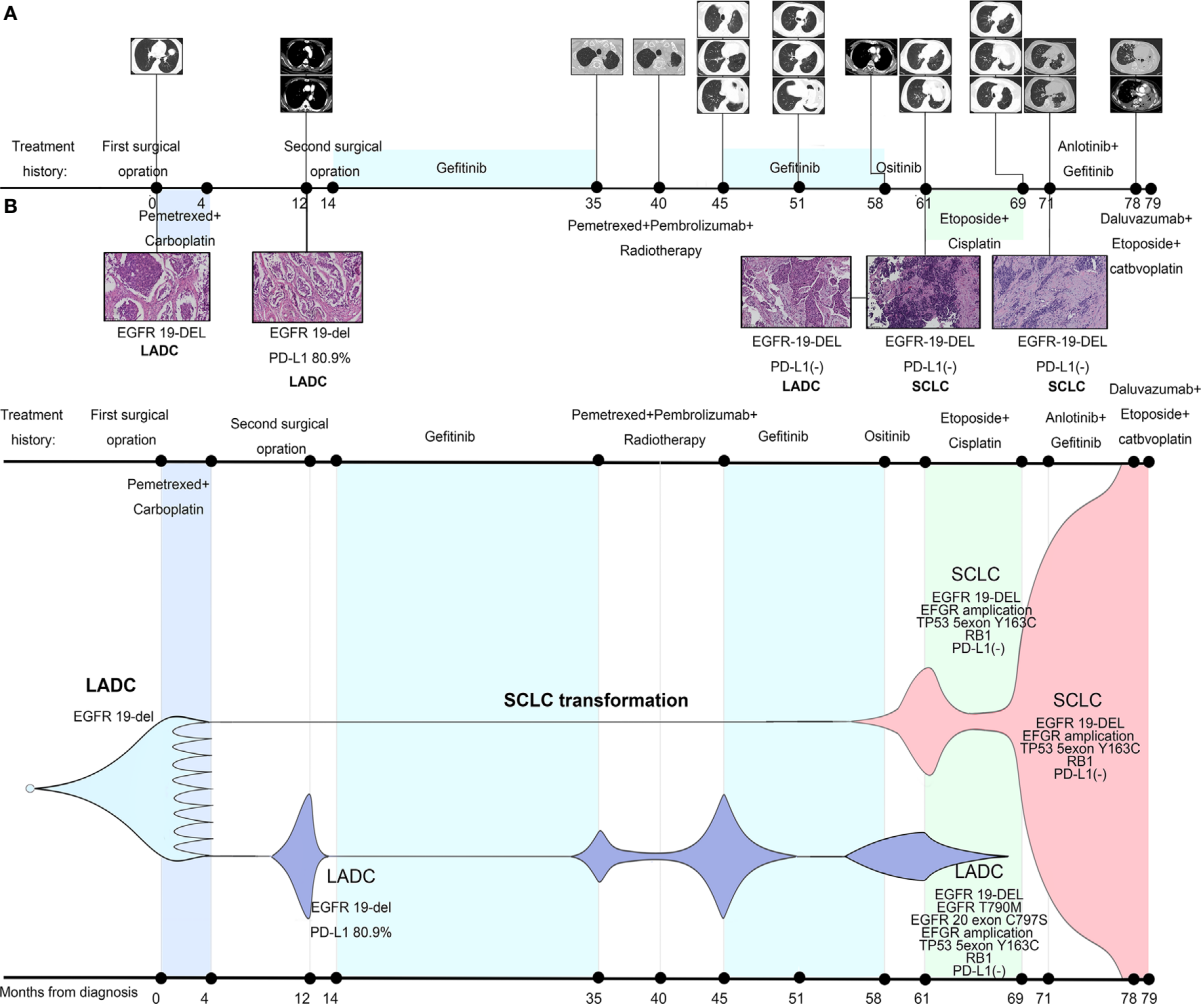
Treatment history of our case and schematic diagram of tumor evolution. **(A)** Clinical treatment history and gene tests results of the patient. Numbers indicate time (in months) from the diagnosis of lung adenocarcinoma (LADC). Scale bar in histopathologic picture indicates 100 μm. **(B)** Presumed clonal evolution of our case which refers to Lee et al.’s study (3). The horizontal axis suggests the clinical history, and the vertical axis represents tumor volume.

A 43-year-old male Asian non-smoker underwent surgical resection of lung mass, with a diagnosis of stage IIA lung adenocarcinoma (pT2bN0M0) harboring an EGFR del-19 mutation and high PD-L1 (80.9%) expression in August 2013 (**Figure 1A**). One year later, he received a second operation for metastasis of mediastinal lymph node and left chest wall. Following that, gefitinib was given for 21 months until clinical resistance when a new nodule in the left residual lung appeared. But genetic testing specimens could not be obtained because it was difficult to have an operation and puncture. Thus, radiotherapy was administrated to control the local tumor. Considering highly expression of PD-L1 in this EGFR TKI resistance patient, eight cycles of pembrolizumab plus pemetrexed were offered, with best response of partial response (PR). The disease progressed again with new metastatic nodules in both lungs. But the multiple lesions were too small to perform a repeated biopsy. Meantime, the patient refused the liquid biopsy. He re-challenged gefitinib for 13 months by himself while drastic response was obtained as PR. When there was a sign of lymphadenopathy recurrence, osimertinib was taken by himself without any clear response. Three months later, the patient was hospitalized due to severe cough and dyspnea. Bronchoscopy was performed and repeated biopsy showed the left lung mass transformed into SCLC while the right remained adenocarcinoma harboring EGFR T790M and cis-C797S mutation in August 2018. Meanwhile, both sides shared the same trunk gene mutations as EGFR 19-del mutation, TP53 and RB1 mutation, with PD-L1 expression changing into negative in both sides. The patient sequentially received etoposide and cisplatin, anlotinib plus gefitinib before multiple metastasis burst out, when biopsies and gene analysis indicated pure SCLC. Finally, he died after one cycle of etoposide and carboplatin plus durvalumab treatment in March 2020.

## Discussion

Patients with a co-occurrence of high PD-L1 (>50%) and EGFR aberrations were disclosed less than 10% in NSCLC (4). A Japanese retrospective study found PD-1 inhibitors were more beneficial as second-line or later treatment for EGFR-TKI resistant NSCLC patients with high PD-L1 expression (TPS>50%) than patients with low PD-L1 expression (5). At the time of resistance to gefitinib, we offered the intervening treatment immunity-combined chemo-radiotherapy to our patient. Radiotherapy could better control the occurrence and development of local lung lesion. Chemotherapy and immunotherapy could diminish much further the clinical and subclinical tumor by activing anti-tumor immunity. Our patient with EGFR-TKI resistance responded well to IO combination therapy, which was consistent with the previous study (5). Thus, PD-L1 TPS 50% or higher may function a predictive role in immunotherapy efficacy after the kinase inhibitor resistance.

However, some patients evolved inevitably IO resistance due to immunosuppressive tumor microenvironment (Treg and MDSCs, VEGF) and immuno-adaption (2) after which the treatment strategies were lacking. In our case, after sequential resistance to gefitinib and pembrolizumab, the patient re-challenged geifitinib by himself. Although, Metro et al. reported that osimertinib re-challenge after chemotherapy in an EGFR T790M‐positive NSCLC patient was feasible and effective (6). The safety was still uncertain because a research suggested osimertinib immediately after nivolumab increased interstitial lung disease incidence in patients with EGFR+ NSCLC (7). Fortunately, this patient did not present any serious AEs, receiving partial response and stale disease for 16 months since re-challenge of the 1^st^ EGFR-TKI after pembrolizumab resistance. In addition, another study also described 1^st^ or 2^nd^ EGFR TKI immediately after nivolumab was safe and effective (8). As such, we speculated that at least for some patients, re-challenge of the 1^st^ EGFR-TKI may be a feasible and safe way to overcome pembrolizumab resistance in EGFR-TKI resistant patients. The underlying mechanism of gefitinib re-sensitization may be that EGFR-TKI drug-resistant tumor cells were lost in the course of chemotherapy, Then in turn, the TKI-sensitive tumor cells re-grow and re-sensitize to the inhibitor (6). Meanwhile, gefitinib re-sensation may contribute to the relapse of previous EGFR+ tumor cells because of T cell exhaustion after ICI therapy resistance. There might be a synergistic effect of immunity-combined chemo-radiotherapy to get gefinitib re-sensitive. It was a process of tumor cells’ evolution due to tumor heterogeneity. Different treatment methods lead to different tumor sub-clone dominant, so diversity of therapy strategies allows patients to afford durable clinical benefits. But, further investigation is warranted.

After the TKI-IO combination therapy-TKI treatment, the case eventually became fully resistant to TKI. As Dr. Robert A. Gatenby’s game theory illustrated in 2018 in JAMA Oncology (9), under continuous treatment pressure, cancer cells inevitably evolved towards treatment escape and malignancy increase. In our case, on the one hand, genetic testing found p.T790M and C797S in cis mutations in the right bronchus. On the other hand, the tumor histologically transformed into SCLC in the left bronchus. In accordance to the study of Lee et al. (3), repeated biopsies of our patient and genetic tests also revealed clearly during 7 years’ survival how SCLCs clone evolve dynamically early from the LADC clones under treatment pressure, eventually leading to SCLC phenotype evading anti-cancer therapy (**Figure 1B** and **Supplementary Table 1**). Furthermore, some previous studies indicated that SCLC transformation only occurred in EGFR-TKI resistant tumors or in LADC without EGFR mutation after ICI therapy resistance (10, 11). Our case is the first to report that SCLC transformation can be achieved after gefitinib–pembrolizumab–gefitinib resistance in EGFR+ NSCLC, providing a new condition for SCLC transformation.

## Data Availability Statement

The original contributions presented in the study are included in the article/**Supplementary Material**, further inquiries can be directed to the corresponding authors.

## Ethics Statement

The studies involving human participants were reviewed and approved by the Institutional Review Board of West China Hospital, Sichuan University. The patients/participants provided their written informed consent to participate in this study. Written informed consent was obtained from the individual(s) for the publication of any potentially identifiable images or data included in this article.

## Author Contributions

XZ and JL wrote the original draft. ZuL and ZhL collected the data and edited the manuscript. YL and LH prepared the figures and reviewed the literature. WW supervised, provided the resource, and reviewed the article. FL conceived the idea and reviewed the article. All authors contributed to the article and approved the submitted version.

## Funding 

This work was supported by the Wu Jieping Medical Foundation, China (312150082).

## Conflict of Interest

The authors declare that the research was conducted in the absence of any commercial or financial relationships that could be construed as a potential conflict of interest.
