# Supplementary material for: Case Report: Re-Sensitization to Gefitinib in Lung Adenocarcinoma Harboring EGFR Mutation and High PD-L1 Expression After Immunotherapy Resistance, Which Finally Transform Into Small Cell Carcinoma
Source: Front Oncol. 2021 Jun 24;11:661034. doi: 10.3389/fonc.2021.661034 (PMC8264361; doi:10.3389/fonc.2021.661034)
Supplement: Supplementary file 1 [file Table_1.pdf]

Supplementary table 1: Gene mutation profiling of the patient as the treatment timeline developed.

**Timing of testing**	**Type of test**	**Sample**	**EGFR 19DEL p.E746 _A750 del**	**EGFR 20 exon p.T790M**	**EGFR 20 exon p.C797S （cis）**	**EGFR amplify-cation**	**PD-L1**	**TP53 exon5 p.Y163C**	**FGF4 exon2 p.S119L**	**RB1 exon 8 splice site mutati on**	**ASXL1 exon 12 p.S993I**	**SPEN exon 11 p.W1340 L**	**GNAS exon 1 p.R685 H**	**PTPRD exon 37 p.I1443**	**KMT2D exon 31 p.R2460 H**
Diagnosis of LADC	qPCR	Lung tissue	(+)				/								
Second surgery	qPCR	Lung tissue	(+)				89%								
Ositinib resistance in right bronchus	NGS 520	Lung tissue	36.78%	46.38%	11.98%	CN=3.38	(-)	20.06%	/	41.13%	18.48%	10.32%	/	/	9.34%
Transform into SCLC in left bronchus	NGS 520	Lung tissue	50.90%	/	/	CN=3.02	(-)	61.30%	7.22%	74.26%	43.99%	21.72%	20.34%	18.72%	/

SCLC progression after EP	NGS 168	Plasma cfDNA	1.18%	/	/	/	/	1.73%	0.53%	2.54%	/	/	/	/	/
SCLC progression after anlotinib plus gefitinib	NGS 168	Lung tissue	38.62%	/	/	/	/	66.95%	25.64%	68.82%	/	/	/	/	CN=3.2
	NGS 168	Plasma cfDNA	13.97%	/	/	/	/	19.35%	0.51%	29.55%	/	/	/	/	CN=2.6
Extensive SCLC	NGS 168	Plasma cfDNA	9.67%	/	/	/	/	12.79%	1.08%	22.12%	/	/	/	/	
